# A yeast model for the mechanism of the Epstein-Barr virus immune evasion identifies a new therapeutic target to interfere with the virus stealthiness

**DOI:** 10.15698/mic2017.09.590

**Published:** 2017-08-31

**Authors:** María José Lista, Rodrigo Prado Martins, Gaelle Angrand, Alicia Quillévéré, Chrysoula Daskalogianni, Cécile Voisset, Marie-Paule Teulade-Fichou, Robin Fåhraeus, Marc Blondel

**Affiliations:** 1 Institut National de la Santé et de la Recherche Médicale UMR1078; Université de Bretagne Occidentale, Faculté de Médecine et des Sciences de la Santé; Etablissement Français du Sang (EFS) Bretagne; CHRU Brest, Hôpital Morvan, Laboratoire de Génétique Moléculaire, 22 avenue Camille Desmoulins, F-29200 Brest, France.; 2 Cibles Thérapeutiques, Institut National de la Santé et de la Recherche Médicale UMR1162, Institut de Génétique Moléculaire, Université Paris 7, Hôpital St. Louis, 27 rue Juliette Dodu, F-75010 Paris, France.; 3 Chemistry, Modelling and Imaging for Biology, CNRS UMR9187 - Inserm U1196, Institut Curie, Université Paris-Sud, Orsay, Campus universitaire, Bat. 110, F-91405, France.

**Keywords:** Epstein-Barr virus (EBV), EBV-related cancers, EBV immune evasion, yeast model for EBV stealthiness, nucleolin (NCL

## Abstract

The oncogenic Epstein-Barr virus (EBV) evades the immune system but has an Achilles heel: its genome maintenance protein EBNA1. Indeed, EBNA1 is essential for viral genome replication and maintenance but also highly antigenic. Hence, EBV evolved a system in which the glycine-alanine repeat (GAr) of EBNA1 limits the translation of its own mRNA at a minimal level to ensure its essential function thereby, at the same time, minimizing immune recognition. Defining intervention points where to interfere with EBNA1 immune evasion is an important step to trigger an immune response against EBV-carrying cancers. Thanks to a yeast-based assay that recapitulates all the aspects of EBNA1 self-limitation of expression, a recent study by Lista *et al.* [Nature Communications (2017) 7, 435-444] has uncovered the role of the host cell nucleolin (NCL) in this process via a direct interaction of this protein with G-quadruplexes (G4) formed in GAr-encoding sequence of EBNA1 mRNA. In addition, the G4 ligand PhenDC3 prevents NCL binding on EBNA1 mRNA and reverses GAr-mediated repression of translation and antigen presentation. This shows that the NCL-EBNA1 mRNA interaction is a relevant therapeutic target to unveil EBV-carrying cancers to the immune system and that the yeast model can be successfully used for uncovering drugs and host factors that interfere with EBV stealthiness.

Yeast-based chemobiological models for a number of human diseases involving functionally conserved physiopathological factors have been quite largely and successfully developed for about fifteen years. However, yeast assays based on the expression of key physiopathological players that do not exist in yeast are also possible (e.g.: yeast models for Huntington and Parkinson diseases). Here we briefly describe how we developed a yeast model to identify drugs and host cell factors that interfere with the capacity of the Epstein-Barr virus (EBV) to evade the host immune system. EBV is the first oncogenic virus discovered in human and has been linked to various cancers that include Burkitt and Hodgkin lymphomas, the nasopharyngeal carcinoma and 10% of gastric cancers. EBV evades the host immune system but, fortunately, has an Achilles heel: its genome maintenance protein EBNA1 which is, on the one hand, essential for EBV genome replication and maintenance and as such expressed in all dividing EBV-infected cells. On the other hand, EBNA1 is highly antigenic and CD8^+^ T cells directed towards EBNA1 epitopes exist in all infected individuals. Hence, in order to avoid the immune system to detect and kill EBV-infected cells, the virus has evolved a mechanism to limit EBNA1 production to the minimal level to sustain viral genome replication and maintenance and, at the same time, to minimize the production of EBNA1-derived antigenic peptides. The central glycine-alanine repeat (GAr) of EBNA1 plays a critical role in this immune evasion strategy as it inhibits the translation of its own mRNA *in cis*, thereby limiting the production of EBNA1-derived antigenic peptides (Fig. 1A). This inhibitory effect of GAr is length-dependent as a longer domain displays a stronger inhibitory effect on both mRNA translation and antigen presentation. Of note, a polymorphism in the length of GAr exists, which may reflect a way for EBV to adapt to its host. GAr-based EBNA1 immune evasion has been considered a relevant therapeutic target to treat EBV-related cancers, hence there is a need to develop cell-based assays that allow high throughput screenings. As the mechanism of GAr-mediated mRNA translation suppression *in cis* was not known, nor the cellular factors involved, a yeast (*Saccharomyces cerevisiae*) assay that recapitulates all the aspects of the GAr-based inhibition of translation, including the GAr-length dependency, has been developed. Briefly, this assay is based on the construction of yeast strains that express individually fusions between GAr of various lengths and the Ade2p reporter protein as sole source of Ade2p enzyme. Yeast cells which express Ade2p at a functional level form white colonies, whereas cells that do not express Ade2p readily form red colonies and cells that express any intermediate level of Ade2p form pink colonies whose coloration intensity is inversely proportional to the level of Ade2p expressed. Because GAr is also able to self-limit in a length-dependent manner the translation of its own mRNA in yeast, this leads to a reduction in Ade2p level which is proportional to GAr size and can be easily monitored by looking at the color of the yeast colonies. A yeast strain that endogenously expressed a fusion between a 43 amino acids GAr and Ade2p was chosen because it leads to a partial inhibition of Ade2p expression, thus leading to pink colonies expressing an intermediate level of Ade2p. Hence, this *43GAr-ADE2* strain allows to look for modifiers (drugs or cellular factors) that either suppress or exacerbate the inhibitory effect of GAr on translation (Fig. 1B). This *43GAr-ADE2* strain was successfully used to identify small molecular weight compounds that can stimulate 43GAr-Ade2p expression in yeast, hence leading to whiter colonies that express higher level of 43GAr-Ade2 (Fig. 1C) whereas having no effect on the control strain expressing Ade2p alone (Fig. 1D). This yeast model for EBNA1 stealthiness was validated since these drugs, which include doxorubicin, were later shown to also stimulate EBNA1 expression in EBV-infected human cells and to relieve GAr-based limitation of antigen presentation. These results also suggested that potential host cell genes involved in EBV immune evasion were conserved in yeast.

**Figure 1:**
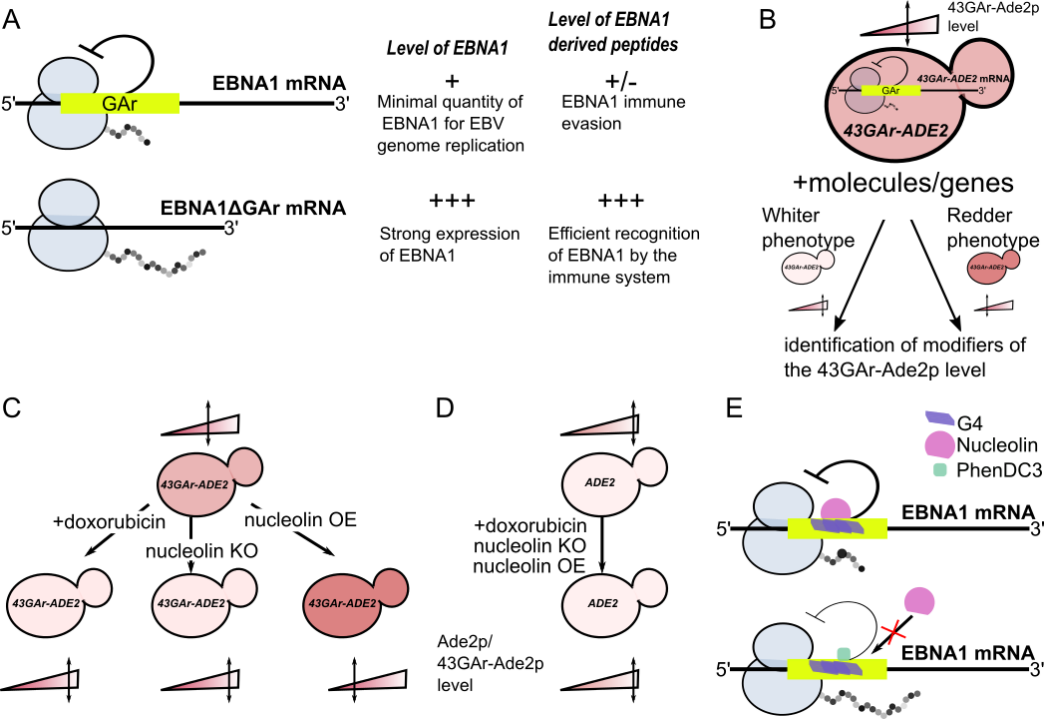
FIGURE 1: Principle of the yeast model for EBNA1 stealthiness and identification of nucleolin as a host factor involved in this process. **(A)** Schematic representation of the GAr effect in self-limitation of EBNA1 mRNA translation. Thanks to the GAr domain, EBNA1 mRNA is translated at the minimal level to allow EBNA1 protein to fulfill its essential function in EBV genome replication and maintenance and, at the same time, to limit production of EBNA1-derived antigenic peptides. The deletion of GAr leads to a strong production of both EBNA1ΔGAr protein and EBNA1-derived antigenic peptides leading to its detection by the immune system. **(B)** Rationale of the yeast model for GAr-dependent inhibition of translation. As GAr is also operant in yeast, the 43GAr-Ade2p fusion protein is only weakly expressed in a strain where it is the only source of Ade2p, thereby leading to the formation of pink colonies that express a limited amount of the protein as compared to a control strain expressing a higher Ade2p level, thus forming white colonies. Hence, based on changes in the intensity of its pink color, this strain can be used to look for modifiers that either suppress or exacerbate the GAr inhibitory effect on translation. **(C)** This 43GAr strain was used for both drug and genetic screening, leading to the isolation of doxorubicin which leads to a GAr-dependent increase in translation and to nucleolin which is a host factor involved in the GAr inhibitory effect on translation. **(D)** The effect of both doxorubicin and nucleolin are GAr-dependent since they have no effect on the control strain expressing Ade2p. **(E)** Nucleolin (NCL) role in EBNA1 immune evasion involves its ability to bind to G4 formed in the GAr-encoding sequence of the EBNA1 mRNA. PhenDC3, a known G4 ligand, binds efficiently GAr’s G4, thereby preventing NCL binding, which results in loss of GAr inhibitory effect on both translation and antigen presentation.

Hence, Lista *et al.* (2017) took advantage of the same yeast assay and of a multicopy plasmid-based yeast genomic library to identify these host cell genes involved in the GAr-mediated inhibition of translation. This way, the yeast *NSR1* gene, which encodes the orthologue of human nucleolin (NCL), was isolated. Indeed, when overexpressed in the pink *43GAr-ADE2* strain, Nsr1p led to the formation of redder yeast colonies that express even less 43GAr-Ade2p, whereas the knockout of the *NSR1* gene (which is viable in yeast) in the same strain led to white colonies that express high level of 43GAr-Ade2p, showing that Nsr1p is involved in GAr-based limitation of protein expression (Fig. 1C). The effect of *NSR1* is GAr-dependent since its overexpression or its inactivation had no effect on Ade2p expression in the control strain (Fig. 1D). Importantly the human NCL was able to complement the *43GAr-ADE2 nsr1Δ* strain that then turned back to its initial pink color and expressed lower levels of 43GAr-Ade2p. Therefore, based on these yeast results, the role of NCL in EBNA1 immune evasion was deciphered in EBV-infected human cells, leading to the formal demonstration that human NCL is involved in GAr-based limitation of translation and antigen presentation.

NCL was known to interact with G-quadruplexes (G4) which are non-canonical secondary structures of guanine-rich nucleic acids formed by the stacking of guanine-quartets which correspond to a planar arrangement of four guanines connected by hydrogen bonds. In the study highlighted here, it was shown that NCL binds directly to the G4s formed in the GAr-encoding sequence of EBNA1 mRNA and that this interaction is crucial for EBNA1 self-limitation of translation and antigen presentation. Importantly, this interaction is druggable since the G4 ligand PhenDC3 prevents NCL from binding to G4 formed in the GAr mRNA sequence of EBNA1 mRNA and stimulates GAr-limited translation and antigen presentation (Fig. 1E). Hence, NCL, the first host cell factor involved in EBNA1 immune evasion, has been uncovered thanks to the yeast model for EBNA1 stealthiness. In addition, this led to the discovery of a pertinent and druggable therapeutic target to trigger an immune response against EBV-related cancers: the interaction between NCL and the G4s of GAr-encoding sequence of the EBNA1 mRNA. Therefore, yeast-based chemo¬biological models for diseases involving key physiopathological actors that do not exist in yeast are definitively worth a trial as, should they work, they potentially allow to easily setup drug and genetic screenings aiming at uncovering both candidate drugs and new therapeutic targets. Of note, doxorubicin, which has been isolated thanks to the yeast assay, has recently been shown to interact with G4, hence potentially filling the gap with nucleolin.

